# Association between functional network connectivity, retina structure and microvasculature, and visual performance in patients after thalamic stroke: An exploratory multi‐modality study

**DOI:** 10.1002/brb3.3385

**Published:** 2024-01-17

**Authors:** Chen Ye, William Robert Kwapong, Biqiu Tang, Junfeng Liu, Wendan Tao, Kun Lu, Ruosu Pan, Anmo Wang, Lanhua Liao, Tang Yang, Le Cao, Youjie Wang, Shuai Jiang, Xuening Zhang, Ming Liu, Bo Wu

**Affiliations:** ^1^ Department of Neurology West China Hospital, Sichuan University Chengdu China; ^2^ Center of Cerebrovascular Diseases West China Hospital, Sichuan University Chengdu China; ^3^ Department of Radiology, Huaxi MR Research Center (HMRRC) West China Hospital, Sichuan University Chengdu China

**Keywords:** brain network, functional connectivity, SS‐OCT/OCTA, thalamic stroke, visual performance

## Abstract

**Background and objective:**

Neuro‐ophthalmologic symptoms and retinal changes have been increasingly observed following thalamic stroke, and there is mounting evidence indicating distinct alterations occurring in the vision‐related functional network. However, the intrinsic correlations between these changes are not yet fully understood. Our objective was to explore the altered patterns of functional network connectivity and retina parameters, and their correlations with visual performance in patients with thalamic stroke.

**Methods:**

We utilized resting‐state functional MRI to obtain multi‐modular functional connectivity (FC), and optical coherence tomography‐angiography to measure various retina parameters, such as the retinal nerve fiber layer (RNFL), ganglion cell‐inner plexiform layer (GCIPL), superficial vascular complex (SVC), and deep vascular complex. Visual acuity (VA) was used as a metric for visual performance.

**Results:**

We included 46 patients with first‐ever unilateral thalamic stroke (mean age 59.74 ± 10.02 years, 33 males). Significant associations were found between FC of attention‐to‐default mode and SVC, RNFL, and GCIPL, as well as between FC of attention‐to‐visual and RNFL (*p* < .05). Both RNFL and GCIPL exhibited significant associations with FC of visual‐to‐visual (*p* < .05). Only GCIPL showed an association with VA (*p* = .038). Stratified analysis based on a disease duration of 6 months revealed distinct and significant linking patterns in multi‐modular FC and specific retina parameters, with varying correlations with VA in each subgroup.

**Conclusion:**

These findings provide valuable insight into the neural basis of the associations between brain network dysfunction and impaired visual performance in patients with thalamic stroke. Our novel findings have the potential to inform future targeted and individualized therapies. However, further comprehensive studies are necessary to validate our results.

## 1 INTRODUCTION

The thalamus is connected to a variety of brain regions relevant to visual functions (Leszczynski et al., 2021; Saalmann & Kastner, 2011). Notably, accounting for 11% of all post‐circulation strokes, neuro‐ophthalmologic symptoms after thalamic strokes have been increasingly noticed (Ivan Adamec et al., 2011; Kim et al., 2015; Lee et al., 2016). Observations from a 10‐year‐long cohort found that 11.7% of the included thalamic stroke patients demonstrated neuro‐ophthalmologic manifestations during the follow‐up, whereas 18.2% among them showed permanent visual‐related deficits, and it is proposed to lead to a great disease burden and serious disability affecting everyday activities (Moon et al., 2021). Detection and understanding of the neural mechanism of brain changes after thalamic stroke is a key target of more precise and effective individualized therapeutic options in the future.

Most previous functional MRI (fMRI) studies only found certain patterns of brain network dysfunction associated with sensorimotor deficits or even vestibular disorders in patients after thalamic stroke (Chen et al., 2019; Conrad et al., 2022; Scharf et al., 2022; Schmahmann, 2003). Actually, these impaired functional networks extend widely within the central nervous system, and some of them are involved with vison processing and transmission structures, including the retina (Usrey & Alitto, 2015). Additionally, increasing evidence has identified alterations in retinal structure and microvasculature obtained by optical coherence tomography‐angiography (OCT/OCTA) in stroke patients, which suggested that the retina can act as a novel and noninvasive imaging marker in cerebrovascular diseases (Cabrera DeBuc et al., 2017; Kwapong, Jiang, et al., 2021; Kwapong, Yan, et al., 2021; Peng et al., 2020; Wang et al., 2014; Zhang et al., 2020). Owing to its connection with the thalamus and the fact that it can be noninvasively visualized, the retina is suggested to provide important information on brain pathology (Jindahra et al., 2010; Xie et al., 2022); and we assume that it could be a future target of imaging in thalamic stroke. Indeed, our previous reports have shown thalamic stroke patients have thinner retinal structures and reduced microvascular densities compared to age‐and‐sex‐matched healthy controls (Ye et al., 2022a, 2022b); importantly, we further showed alterations of structural changes around the optic nerve head and retina in thalamic stroke patients were related to shrinkage of the optic tract, and all these changes were correlated with visual performance (Ye et al., 2022b). Taken together, our previous studies showed that the retinal microvasculature and structural thicknesses (which represents the retinal neural integrity) can mirror cerebral microcirculation and neuronal changes associated with thalamic stroke.

Hence, we aimed to conduct an exploratory investigation in patients after thalamic stroke based on multi‐modality images, that is, fMRI and OCT/OCTA, to explore the linking patterns within the retinal structural and microvascular changes and muti‐modular functional connectivity (FC) and further explor their associations with visual performance. We hypothesize that in thalamic stroke patients, changes in retinal parameters may be associated with cerebral FC parameters.

## 2 METHODS

### 2.1 Participants

**FIGURE 1:**
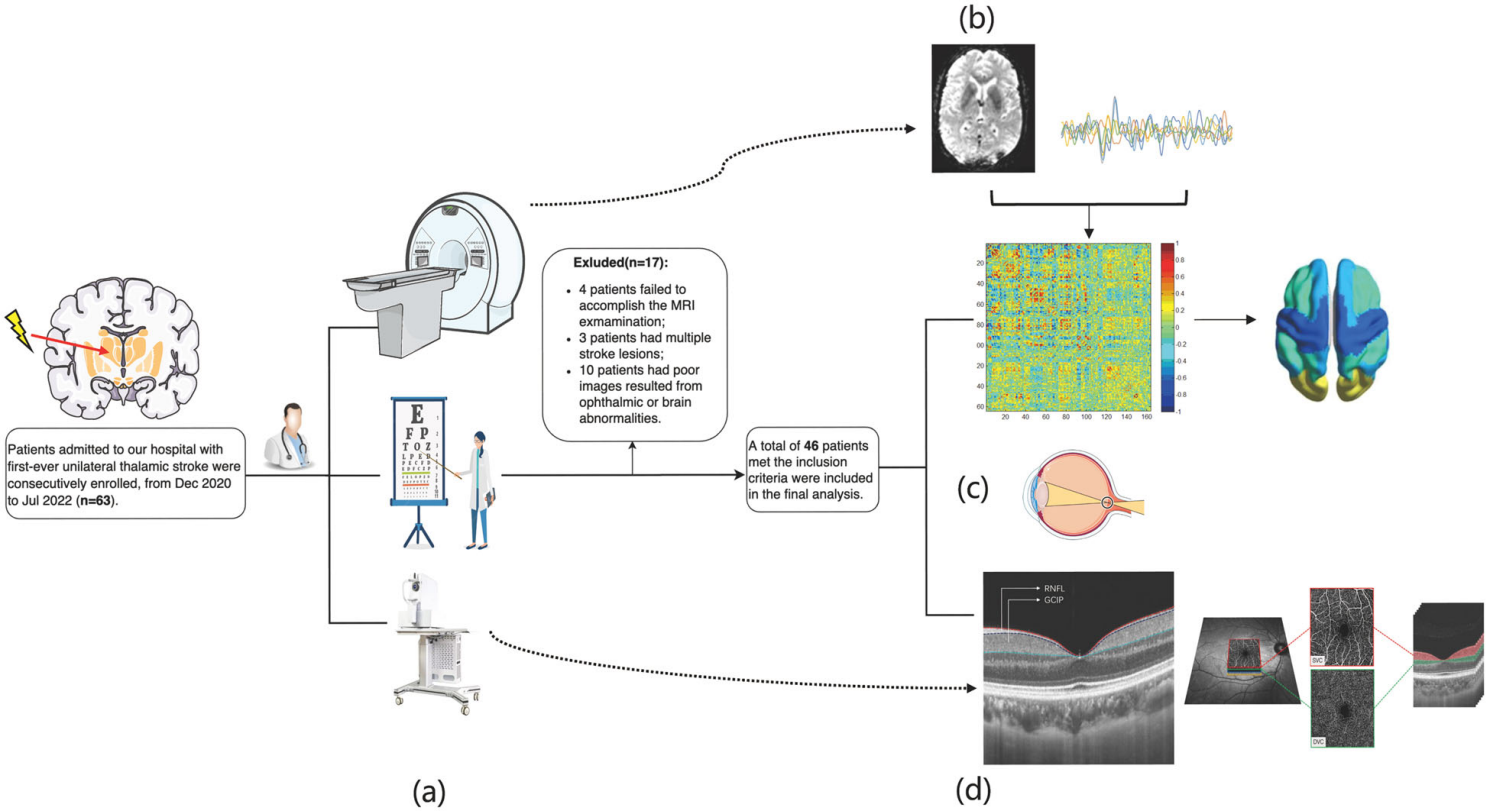
Schematic overview of the analysis pipeline and flowchart of patient enrolments: (a) Flowchart of patient enrolments and clinical evaluation including MRI scanning, swept source‐optical coherence tomography‐angiography (SS‐OCT/OCTA) examining, and visual acuity (VA) test; (b) demonstration of functional MRI (fMRI) time series and functional connectivity (FC) matrices and then divided into modules according to atlas‐based parcellation; (c) visual performance was assessed using VA under illumination and later converted to the logarithm of the minimum angle of resolution (LogMAR); (d) segmentation of retina structure and microvasculature.

Consecutive patients with first‐ever unilateral thalamic stroke were enrolled from the Department of Neurology, West China Hospital of Sichuan University between December 2020 and July 2022. The inclusion and exclusion criteria were detailed in our previous report (Ye et al., 2022b). A systematic flowchart of the study design and participants’ enrollment is shown in Figure 1a. This cross‐sectional study was approved by the Ethics Committee of West China Hospital of Sichuan University [No. 2020 (922)]. All participants gave written informed consent following the Declaration of Helsinki.

Demographic and clinical information were collected in a standardized format, including sex, age, and risk factors for cerebrovascular disease (history of hypertension, diabetes, and dyslipidemia). Time since stroke onset (months) and National Institute of Health Stroke Scale (NIHSS) scores were also documented. Standardized and detailed neurological examinations, including eye movement and visual field, were conducted by two experienced neurologists. Visual performance was assessed using visual acuity (VA) under illumination and later converted to the logarithm of the minimum angle of resolution by an experienced neuro‐ophthalmologist.

Additionally, the stroke lesion volume was measured by drawing lesion masks manually on the structural MR images using MRIcron (Rorden & Brett, 2000) blinded to clinical information. Then, individual lesion masks were normalized to standard Montreal Neurological Institute (MNI) space with isotropic 1 mm^3^ voxels using Statistical Parametric Mapping 12 (Eickhoff et al., 2005), and lesion volumes (cm^3^) were calculated for each participant.

### 2.2 MRI data acquisition

All participants enrolled underwent MRI scanning on a 3.0T MR scanner (SIGNA Premier, GE Healthcare) with the 48‐channel head coil. Imaging data were collected on the same day when clinical and OCT/OCTA assessments were completed. Head motion and scanner noise were reduced using tight but comfortable foam padding and earplugs. All participants were required to lie quietly and remain awake with their eyes closed, trying to avoid mental activities during scanning process. The high‐resolution 3D T1‐weighted and resting‐state blood‐oxygen‐level (BOLD) rs‐fMRI were collected. Besides, routine clinical T1‐weighted, T2‐weighted, fluid‐attenuated inversion recovery, and diffusion‐weighted images sequences were also obtained and conventionally reviewed to exclude obvious structural abnormalities. Two experienced neuroradiologists inspected each scan to exclude the imaging data with visible movement, gross image artifacts, and brain abnormalities that might affect the following preprocessing procedures. All participants in this study met the MRI data inclusion criteria of spatial movement in any direction <3 mm or rotation <3°. The high‐resolution 3D T1‐weighted anatomical images were acquired by brain volume sequence (slices = 156, a field of view = 256 mm × 256 mm, echo time = 3.0 ms, repetition time = 7.2 ms, flip angle = 12°, matrix size = 256 mm × 256 mm, and isotropic voxels size = 1 mm^3^). The resting‐state BOLD fMRI was collected by gradient echo‐planar imaging sequence (slices = 65, a field of view = 208 mm × 208 mm, echo time = 39 ms, repetition time = 1000 ms, flip angle = 90°, matrix size = 86 mm × 86 mm, and isotropic voxels size = 2.4 mm^3^, no gap, a total of 330 volumes).

### 2.3 Resting‐state fMRI data preprocessing

The resting‐state fMRI data were preprocessed using configurable pipeline for analysis of connectomes (http://fcp‐indi.github.com), which is a python‐based pipeline tool involving AFNI (Cox, 1996), ANTs (Tustison et al., 2014), FSL (Jenkinson et al., 2012), and custom python code. The fMRI data were preprocessed as follows: discarding the first 10 volumes to obtain steady‐state magnetization, realignment for head movement, slice‐time correction, and segmentation. The remaining volumes were registered with the individual‐level 3D‐T1 images and then normalized in the standard MNI space with isotropic 3 mm^3^ voxels. Denoising was then using Conn (Whitfield‐Gabrieli & Nieto‐Castanon, 2012) with the implementation of CompCor (Behzadi et al., 2007) by performing principal component analysis on eroded motion parameters, white matter, and cerebrospinal fluid signals with regression of the first five components. Subsequently, linear detrending and bandpass filtering (0.01–0.08 Hz) were performed to reduce the influence of signal drift and high‐frequency noise. Finally, images were spatially smoothed with a Gaussian filter with a full width at half maximum of 6 mm.

### 2.4 Multi‐modular brain functional connectivity calculation

The preprocessed average time series of each node (defined by automated anatomical labeling atlas, AAL template) (Rolls et al., 2020) area were obtained by averaging the voxel BOLD signals in the selected brain area (Tzourio‐Mazoyer et al., 2002). Pearson's correlation coefficient (between the average time series for each node) was computed as the strength of FC, Figure 1b. For further statistical analysis, the correlation coefficients were transformed to *z*‐values using the Fisher *r*‐to‐*z* transformation to improve the normality.

Each of the brain regions (i.e., nodes) divided using AAL was associated with its corresponding resting‐state functional network. Five empirical and robust functional networks (i.e., modules) (He et al., 2009) were further extracted from the resting‐state function networks, consistent with previous research studies (Reber et al., 2021; Tarchi et al., 2022; Xin et al., 2022; Zhang et al., 2022), defined as follows: Sensorimotor network module, visual network module, attention network module, default mode network (DMN) module, and subcortical network module are detailed in Table S1 and Figure S1. The FC within each module was calculated as the average of all connection weights in the module. The inter‐module connection strength between every two modules was also calculated to represent the average of the connection weights connecting the two modules. Thus, the multi‐modular FC within and between modules (i.e., within‐ and inter‐module level) of the resting‐state subnetwork was computed and used for subsequent analysis.

### 2.5 Retinal structure and microvasculature imaging

Swept source OCT (SS‐OCT)/SS‐OCTA tool was used to measure retinal structure and microvasculature parameters, as shown in Figure 1d. SS‐OCT/OCTA (VG 200; SVision Imaging Limited) was used to scan and image the macula of all participants. The structural OCT imaging was done with 18 radial scan lines positioned on the fovea. Each scan line was generated by 2048 A‐scans, was 12 mm long, and was separated from adjacent lines by 10°. Sixty‐four B‐scans were obtained on each scan line and were automatically averaged to improve the signal‐to‐noise ratio (Alonso‐Caneiro et al., 2011). The system was equipped with an eye‐tracking utility based on an integrated confocal scanning laser ophthalmoscope to eliminate eye‐motion artifacts. Automatic segmentation of the retinal structural thickness was done by the OCT tool. Our current study focused on the retinal nerve fiber layer (RNFL, μm) and ganglion cell‐inner plexiform layer (GCIPL, μm) in a 3 × 3 mm^2^ area around the fovea.

The microvascular images were obtained using OCTA fundus imaging tool with a raster scan protocol of 384 horizontal B‐scans that covered an area of 3 × 3 mm^2^ centered on the fovea. The axial resolution, lateral resolution, and scan depth were 5 μm, 13 μm, and 3 mm, respectively. The superficial vascular complex (SVC) and deep vascular complex (DVC) were segmented and generated by in‐built software. The two plexuses were found 5 μm above the inner limiting membrane to 25 μm below the inner nuclear layer (INL). The SVC and DVC were in the inner two‐thirds and outer one‐third of the ganglion cell layer and inner plexiform layer (GCL + IPL), as shown in Figure 1d. The microvascular density which was used to assess the microvasculature of the macular plexuses was defined as the percentage of microvasculature occupied in the annulus region of measurement (3 × 3 mm^2^ centered on the fovea).

All retinal measurements were done at the macula. OCT/OCTA data displayed in our study followed the OSCAR‐IB quality criteria (Tewarie et al., 2012) and APOSTEL recommendation (Aytulun et al., 2021). The quality of the macular images was assessed objectively and subjectively, rejecting images with a signal quality of less than 8 on a scale of 10. En face angiograms with artifacts, blurry images, and the presence of retinal diseases on images such as age‐related macular degeneration, severe cataract, optic neuritis, and macular edema were also excluded.

### 2.6 Statistical analysis

Continuous variables with normal distribution are expressed as mean with standard deviation or medians with interquartile ranges (IQR) where appropriate. Categorical variables are presented as frequencies and percentages (%). Multiple linear regression was used to explore the correlation between OCT and OCTA parameters and FC measures in thalamic stroke patients with age, gender, hypertension, diabetes, dyslipidemia, disease duration, and lesion volume as nuisance variables. Consistent with our previous studies (Ye et al., 2022a, 2022b), stratified analysis was performed based on a disease duration of 6 months since it has been well recognized that the process of *trans*‐retrograde degeneration is time‐dependent (Jindahra et al., 2012), along with the time course of poststroke visual deficit becoming steady after 6 months (Fahrenthold et al., 2021). *p*‐Value less than .05 (*p* < .05) was considered statistically significant. Data analysis and plotting were performed with R version 4.0.3.

## 3 RESULTS

### 3.1 Baseline characteristics

From December 2020 to July 2022, 63 patients with first‐ever and suspected unilateral thalamic stroke visited our hospital and participated in the present study. All patients underwent our full set of comprehensive clinical evaluations and a total of 46 patients were included finally. Seventeen patients were excluded for the reasons as shown in Figure 1. The mean age of the included patients was 59.74 ± 10.02 years old, and 33 were males. The baseline NIHSS (National Institute of Health Stroke Scale) score was 1 (IQR 1–2). The disease duration upon admission was 0.3 months (IQR 1.5–12). Retinal microvasculature and structural parameters, as well as FC within and between five modules (sensorimotor network, visual network, attention network, DMN, and subcortical network), were shown in Table 1.

**TABLE 1:** Baseline clinical information, retinal, and neuroimaging characteristics.

Characteristics	Data (*n* = 46)
**Clinical information**	
Age, years (SD)	59.74 (10.02)
Gender, males	33
SBP, mmHg (SD)	133.63 (20.83)
DBP, mmHg (SD)	82.72 (13.77)
Hypertension, *n*	22
Diabetes, *n*	13
Dyslipidemia, *n*	9
Smoking, *n*	21
Alcohol, *n*	21
NIHSS score (IQR)	1 (1–2)
Lesion location, L/R, *n*	21/25
Stroke subtype, I/H, *n*	44/2
Disease duration, months (IQR)	0.3 (1.5–12)
**Retinal characteristics, (SD)**	
SVC, %	38.32 (6.27)
DVC, %	48.59 (4.43)
RNFL, μm	19.36 (2.17)
GCIPL, μm	73.34 (9.69)
**FC within and between modules, (IQR)**	
Attention to attention	0.386 (0.340–0.403)
Attention to default mode	0.285 (0.255–0.311)
Attention to sensorimotor	0.276 (0.238–0.314)
Attention to subcortical	0.200 (0.178–0.226)
Attention to visual	0.195 (0.162–0.227)
Default mode to default mode	0.393 (0.348–0.431)
Default mode to sensorimotor	0.200 (0.180–0.226)
Default mode to subcortical	0.227 (0.212–0.273)
Default mode to visual	0.209 (0.172–0.259)
Sensorimotor to sensorimotor	0.428 (0.347–0.487)
Sensorimotor to subcortical	0.228 (0.198–0.256)
Sensorimotor to visual	0.214 (0.178–0.261)
Subcortical to subcortical	0.269 (0.241–0.320)
Subcortical to visual	0.167 (0.142–0.201)
Visual to visual	0.516 (0.455–0.678)

Abbreviations: DBP, diastolic blood pressure; DVC, deep vascular complex; FC, functional connectivity; GCIPL, ganglion cell and inner plexiform layer; H, hemorrhagic; I, ischemic; IQR, interquartile range; L, left; NIHSS, National Institute of Health Stroke Scale; R, right; RNFL, retinal nerve fiber layer; SBP, systolic blood pressure; SD, standard deviation; SVC, superficial vascular complex.

### 3.2 Association between multi‐modular FC and retina structural and microvascular characteristics

**FIGURE 2:**
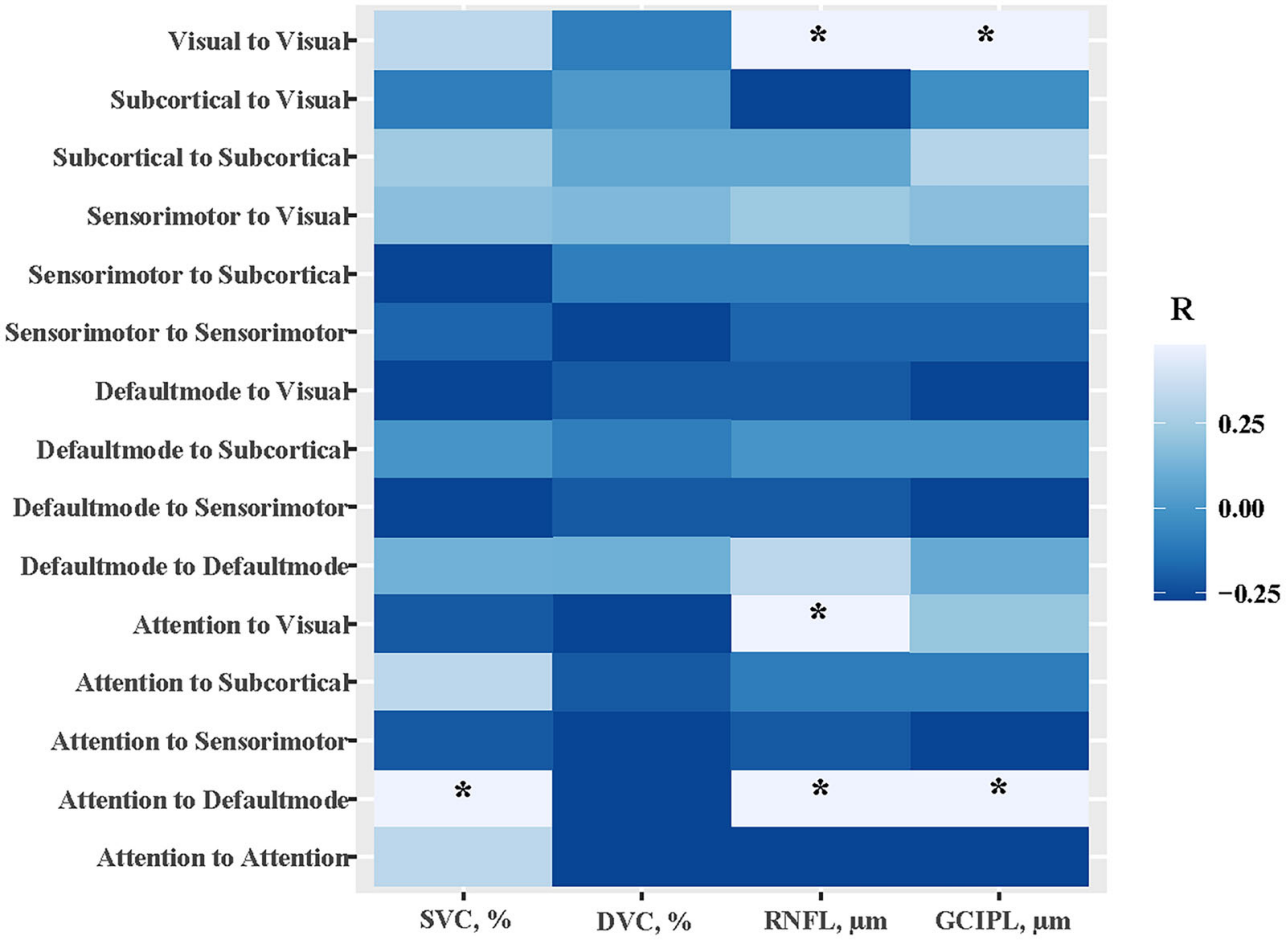
Heatmap of correlations between multi‐modular functional connectivity and retina structural and microvascular characteristics. *Statistically significant with *p* < .05. Adjusted for age, gender, hypertension, diabetes, dyslipidemia, and lesion volume.

We explored the associations between multi‐modular FC and retina parameters, as shown in Figure 2. At the inter‐module level, significant associations were found in FC of attention‐to‐default mode with SVC (*p* = .021), RNFL (*p* = .010), and GCIPL (*p* = .036), and in FC of attention‐to‐visual with RNFL (*p* = .026) after adjustment of nuisance variables (age, gender, hypertension, diabetes, dyslipidemia, disease duration, and lesion volume). At the within‐module level, both RNFL (*p* = .008) and GCIPL (*p* = .005) were significantly and independently associated with the FC of visual‐to‐visual. No significant associations were found in other module levels and retina characteristics, details shown in Table S2.

### 3.3 Association between retina structural and microvascular characteristics and VA

**FIGURE 3:**
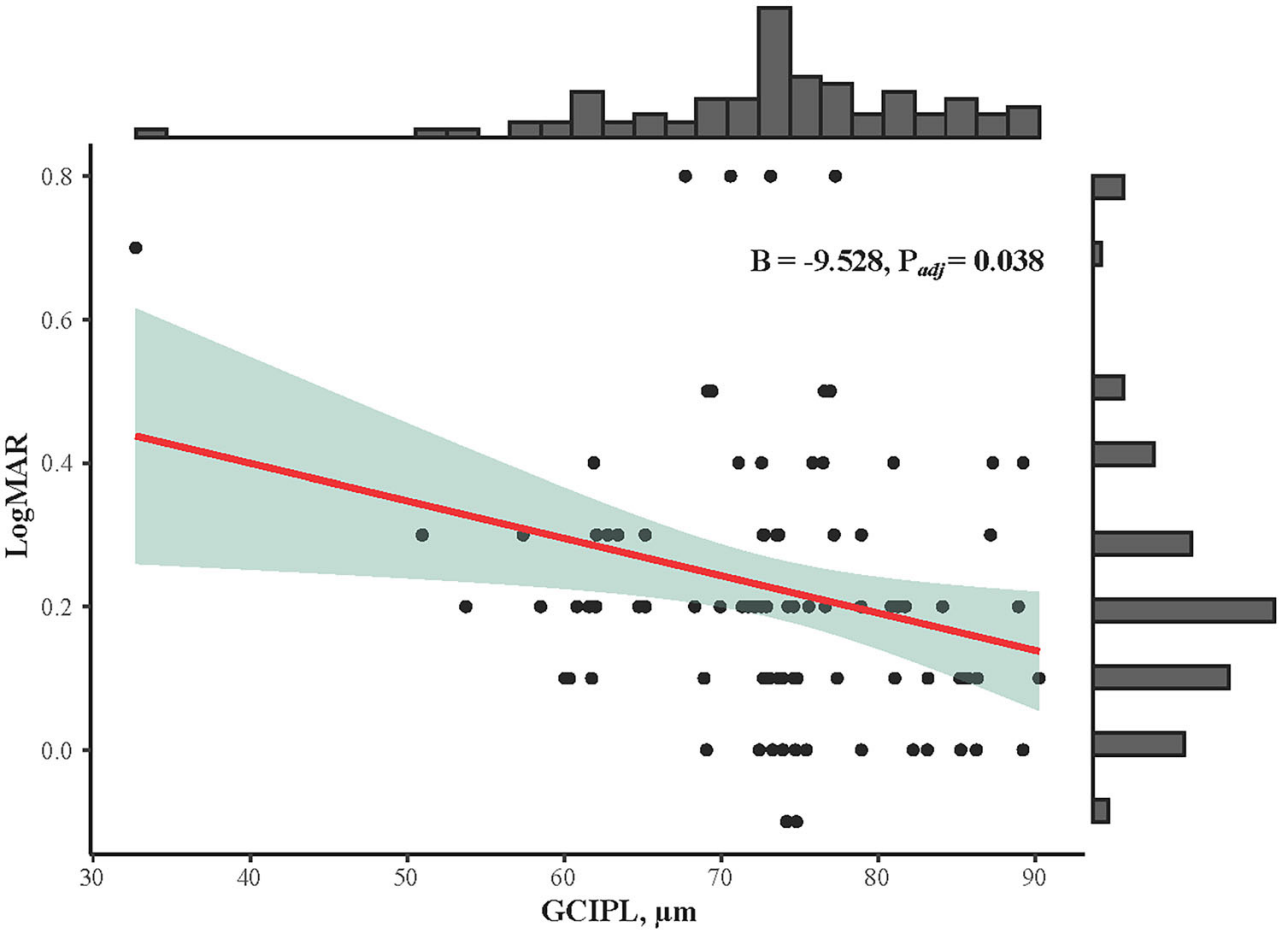
Association between ganglion cell‐inner plexiform layer (GCIPL) and visual acuity (VA), LogMAR. A significant correlation was found between GCIPL and VA (*B* = −9.528, *p* = .038) after adjustment for age, gender, hypertension, diabetes, dyslipidemia, disease duration, and lesion volume. Raw data were plotted with adjusted statistic values.

The associations between retina structural and microvascular characteristics and VA were analyzed, as shown in Table 2 and Figure 3. Only GCIPL was significantly associated with VA (*ß* = −9.528, *p* = .038) after adjustment for age, gender, hypertension, diabetes, dyslipidemia, disease duration, and lesion volume.

**TABLE 2:** Association between retina structural and microvascular characteristics and visual acuity (VA).

	*B*	SE	*p*
SVC	−4.651	3.533	.192
DVC	.141	2.292	.951
RNFL	.131	1.192	.912
GCIPL	−9.528	5.211	.038

*Note*: Adjusted for age, gender, hypertension, diabetes, dyslipidemia, disease duration, and lesion volume.

Abbreviations: DVC, deep vascular complex; GCIPL, ganglion cell and inner plexiform layer; LogMAR, logarithm of the minimum angle of resolution; RNFL, retinal nerve fiber layer; SVC, superficial vascular complex; VA, visual acuity.

### 3.4 Association between multi‐modular FC and VA

The associations between multi‐modular FC and VA were also explored, as shown in Table S2. Neither inter‐module FC nor within‐module FC was found to be significantly associated with VA.

### 3.5 Stratified analysis of patients by disease duration

#### 3.5.1 Analysis in patients with disease duration ≤6‐month

**FIGURE 4:**
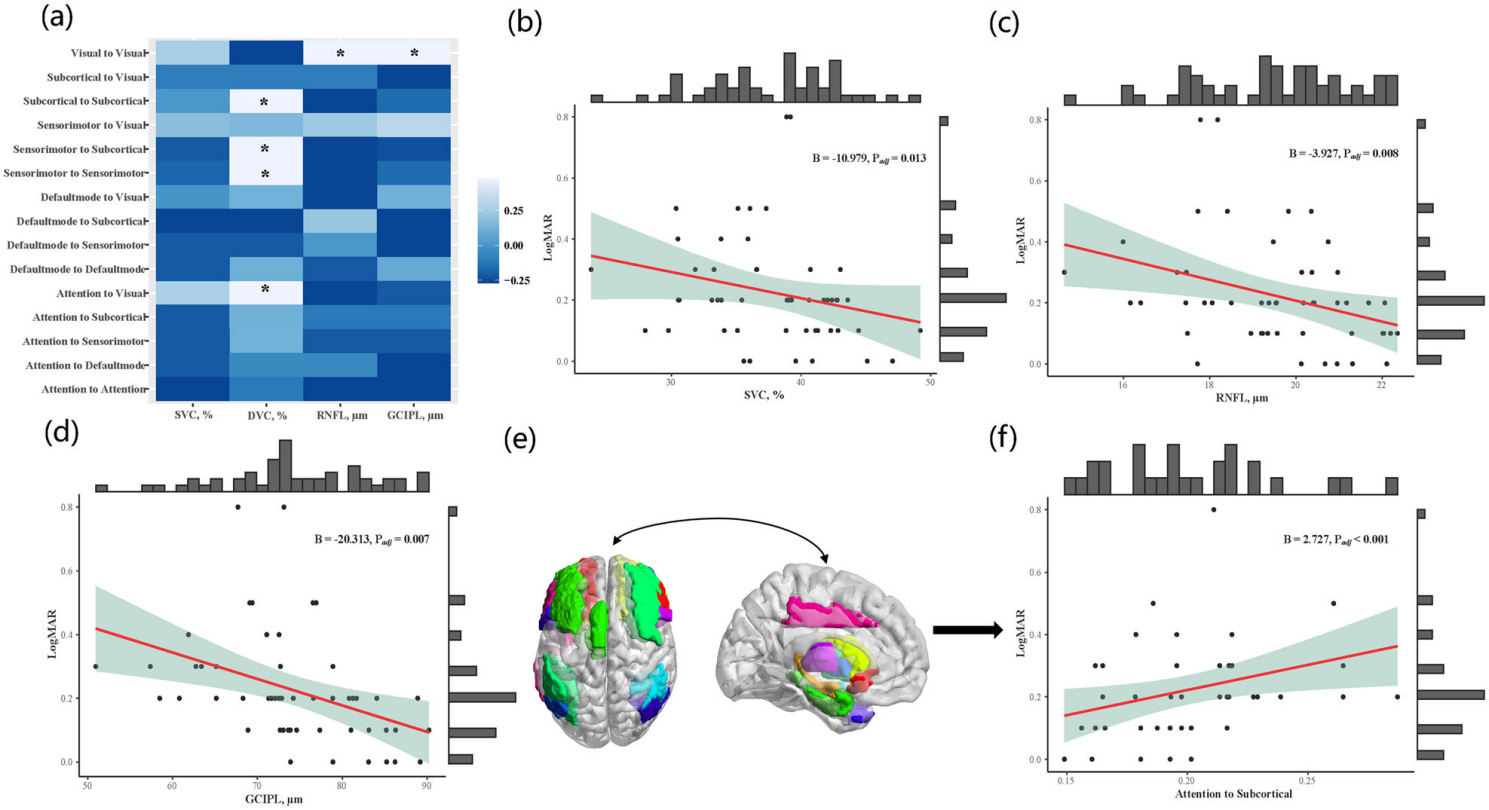
Subgroup analysis in patients with disease duration ≤6‐month. (a) Heatmap of correlations between multi‐modular functional connectivity (FC) and retina structural and microvascular characteristics. *Statistically significant with *p* < .05. (b–d) Significant correlations of superficial vascular complex (SVC) (*B* = −10.979, *p* = .013), retinal nerve fiber layer (RNFL) (*B* = −3.927, *p* = .008), and ganglion cell‐inner plexiform layer (GCIPL) (*B* = −20.313, *p* = .007) with visual acuity (VA) after adjustment for age, gender, hypertension, diabetes, dyslipidemia, and lesion volume. Raw data were plotted with adjusted statistic values. (e and f) FC of attention‐to‐subcortical significantly associated with VA.

As shown in Figure 4a, at the inter‐module level, DVC was significantly associated with FC of attention‐to‐visual (*p* = .040) and FC of sensorimotor‐to‐subcortical (*p* = .014) modules. At the within‐module level, significant associations were found in DVC with FC of subcortical‐to‐subcortical (*p* = .047) and sensorimotor‐to‐sensorimotor (*p* = .006), as well as RNFL (*p* = .020) and GCIPL (*p* = .013) with FC of visual‐to‐visual. Moreover, significant associations of SVC (*ß* = −10.979, *p* = .013), RNFL (*ß* = −3.927, *p* = .008), and GCIPL (*ß* = −20.313, *p* = .007) with VA were also found, shown in Figure 4b–d and Table S4. The associations between multi‐modular FC and VA were further explored and only FC of attention‐to‐subcortical was found to be significantly associated with VA (*ß* = 2.727, *p* < .001), displayed in Figure 4e,f. More details were presented in Tables S3 and S4.

#### 3.5.2 Analysis in patients with disease duration >6‐month

**FIGURE 5:**
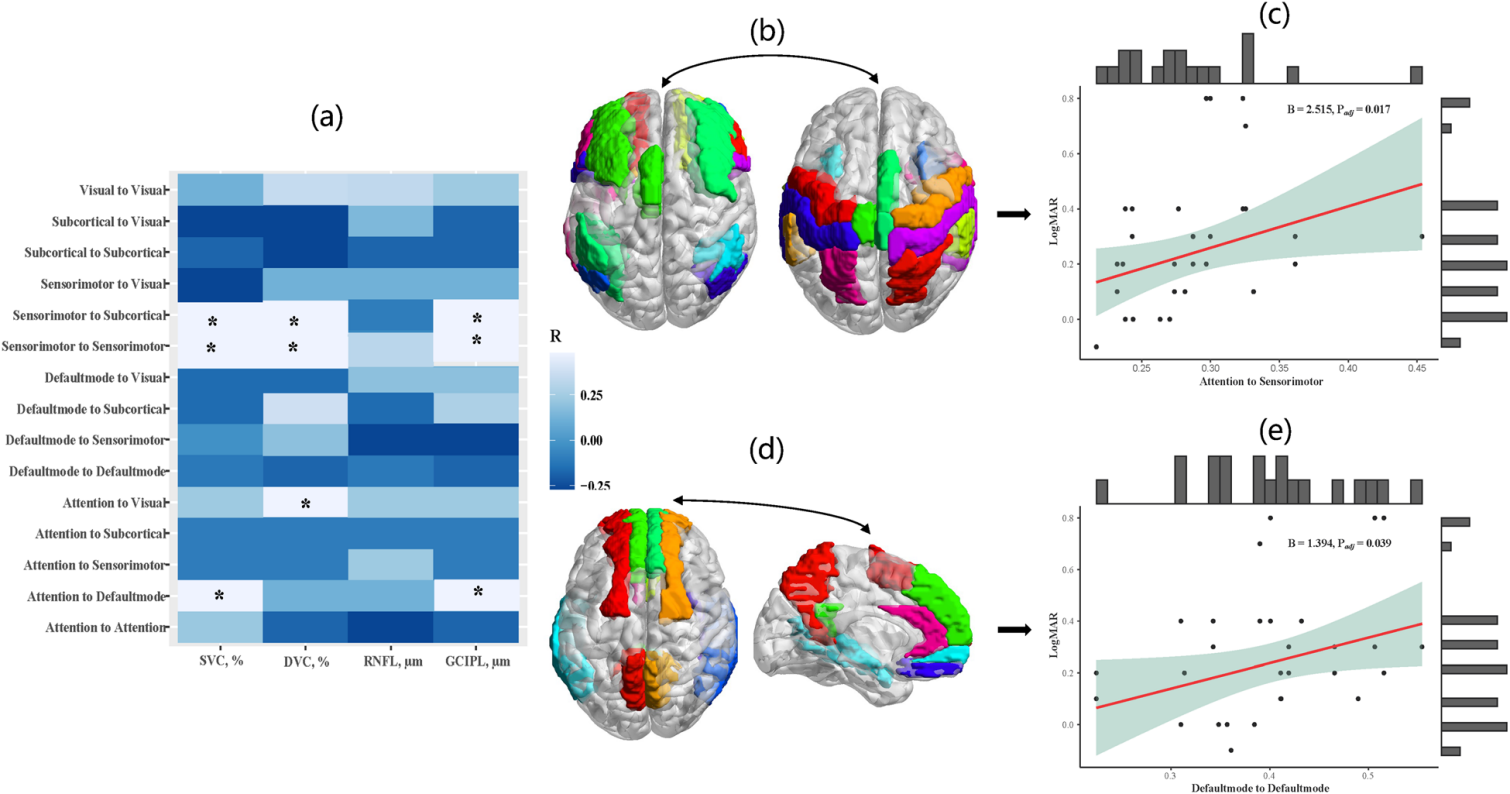
Subgroup analysis in patients with disease duration >6‐month. (a) Heatmap of correlations between multi‐modular functional connectivity (FC) and retina structural and microvascular characteristics. *Statistically significant with *p* < .05. (b and c) FC of attention‐to‐sensorimotor at inter‐module level (*B* = 2.525, *p* = .017) significantly associated with visual acuity (VA). (d and e) FC of default mode‐to‐default mode at within‐module level (*B* = 1.394, *p* = .039) significantly associated with VA. Adjustment for age, gender, hypertension, diabetes, dyslipidemia, and lesion volume. Raw data were plotted with adjusted statistic values.

In the subgroup of disease duration >6‐month, SVC (*p* = .042), DVC (*p* = .025), and GCIPL (*p* = .036) were significantly associated with FC of sensorimotor‐to‐sensorimotor at the within‐module level. Meanwhile, at the inter‐module level, SVC was significantly associated with FC of attention‐to‐default mode (*p* = .008) and sensorimotor‐to‐subcortical (*p* = .035). Similarly, significant associations were found in DVC with FC of attention‐to‐visual (*p* = .003) and sensorimotor‐to‐subcortical (*p* = .002), and GCIPL with FC of attention‐to‐default mode (*p* = .04) and sensorimotor‐to‐subcortical (*p* = .008), shown in Figure 5a and Table S5. No significant association was found between retina and VA (Table S6). Furthermore, the association between FC at multi‐modular levels and VA was also explored, detailed in Table S5. The FC of attention‐to‐sensorimotor at inter‐module level (*ß* = 2.525, *p* = .017) and FC of default mode‐to‐default mode at within‐module level (*ß* = 1.394, *p* = .039) were significantly associated with VA, shown in Figure 5b–e, respectively.

## 4 DISCUSSION

Our current study goes beyond our previous reports (Ye et al., 2022a, 2022b), which suggest that structural and microvascular changes in the retina reflect the cerebral microstructure and microvascular in thalamic stroke patients, conducting a multi‐modality exploratory study to investigate the retinal parameters in thalamic stroke and their association with cerebral multi‐modular FC and visual performance (VA). We showed retinal thicknesses (RNFL and GCIPL) and SVC correlated with FC in thalamic stroke patients. In subgroup with duration ≤6 and >6 months, significant correlations between retinal parameters, VA, and FC at multi‐modular levels were found. These findings suggest that lesioned thalamus may affect the functional reorganization of the whole brain network, and these brain network disorders were correlated with both the retina changes and VA to various extents.

Functional network alterations have been well described in thalamic stroke patients in rs‐fMRI studies, which showed widely reduced FC and suggested that the reduced connectivity may correlate to clinical symptoms (Kim et al., 2013; Liu et al., 2016; Rehme & Grefkes, 2013). Particularly, frequent reduced FC in the DMN module and dorsal attention network were demonstrated in thalamic stroke patients (Bonkhoff et al., 2020; Chen et al., 2019; He et al., 2021). The DMN and attention network are engaged in paying attention (Li et al., 2014), and our study showed that the attention‐to‐default mode module FC correlated with their SVC density and RNFL and GCIPL in thalamic stroke patients. As it is suggested that the reduced microvascular density in the superficial vessels and thinner RNFL and GCIPL thickness may reflect the cerebral changes that occur after thalamic stroke 21 and other cerebral disease (Kwapong, Yan, et al., 2021; Peng et al., 2020), we assume that the associations between FC in attention‐to‐default mode and retina (SVC and RNFL and GCIPL) indicate that microvascular impairment and neurodegeneration might affect their attention and ultimately impact the visual performance.

Reduced vision is one clinical manifestation after thalamic stroke (Gooneratne et al., 2015; Schaller‐Paule et al., 2021; Ye et al., 2022a, 2022b). Our previous report showed that visual loss in thalamic stroke patients is associated with retinal neurodegeneration (Ye et al., 2022b). The RNFL and GCIPL form the retinal ganglion cells play an important role in vision (Rossi et al., 2017); thus, damage to these layers may affect vision. We showed the RNFL correlated with attention‐to‐visual FC while RNFL and GCIPL correlated with visual‐to‐visual FC in thalamic stroke patients; in duration *≤*6‐month group, SVC and RNFL and GCIPL correlated with visual‐to‐visual FC. Taken together, these findings suggest that network alterations are linked with retinal changes ultimately leading to reduced vision, supported by a previous study that showed thalamic stroke patients had reduced visual‐related functional network (Kim et al., 2013).

In this study, GCIPL correlated with their VA, whereas SVC, RNFL, and GCIPL correlated with VA in subgroup of *≤*6‐month duration. Nonetheless, no association was seen in the VA of thalamic stroke patients with duration >6‐month and their retinal parameters. Some studies previously reported relationships between VA and GCIPL that was related to the pathophysiology of the disease (Rashid et al., 2021). We previously showed that thalamic stroke patients with a duration >6‐month had thicker retinal structures compared to patients with a duration *≤*6‐month; we also showed that thinning of the GCIPL thickness was associated with deteriorating vision (Ye et al., 2022b). In current study, as the thickness of the GCIPL reduced, VA in thalamic stroke patients worsened during the early phase (*≤*6‐month). Our finding suggests the importance of early intervention of preventing neuro‐axonal damage and further deterioration, particularly for ganglion‐cell death, for preserving vision.

Located in the INL, DVC consists of bipolar, amacrine, and horizontal cells and is solely made of capillaries (Campbell et al., 2017). We showed that the DVC in thalamic stroke patients with a duration *≤*6‐month or more than 6‐month showed associations with FC. Our findings suggest that deeper microvasculature is linked with connectivity changes in thalamic stroke patients when stratified according to their duration. To the best of our knowledge, this is the first study to report on these associations; future studies are needed to warrant our speculations.

Our study has some limitations. Even though our results showed significant associations between cerebral FC and retinal parameters and VA in thalamic stroke patients, our cross‐sectional design does not allow the explanation of the underlying causal influence. Another limitation is the small sample size in our study; besides, no symptoms of the visual field or oculomotor deficits occurred, which diminished the clinical interest and expandability to some extent. However, as a type of ischemic stroke with a lower proportion, only 11.7% of thalamic insulted patients developed prominent neuro‐ophthalmologic manifestations in a 10‐year cohort study (Moon et al., 2021). Long‐term follow‐up cohort studies with clinical evaluations on dynamic changes and larger sample sizes with comprehensive neuro‐ophthalmologic characteristics are needed in the future. Finally, field maps were not collected for further corrections. In the present study, rigorous scanning and data processing procedures were adopted to ensure image quality. In subsequent studies we will include the field map scanning sequence to ensure more robust results.

In conclusion, to the best of our knowledge, this is the first study to explore the association between FC changes at a multi‐modular level in thalamic stroke patients and retinal parameters and their VA. We showed distinct cerebral FC changes at diverse modularity in thalamic stroke patients are associated with certain retinal structural and microvascular changes and their VA. Taken together, our study suggests that a combination of OCT/OCTA, cerebral imaging modalities, and clinical features in thalamic stroke patients can be used to elucidate the pathophysiology of visual impairment that occurs in most thalamic stroke patients. Longitudinal studies with larger sample sizes are needed to warrant our speculations and recognize possible insinuations for treatment in patients with thalamic stroke.

## AUTHOR CONTRIBUTIONS

**Chen Ye**: Conceptualization; methodology; software; data curation; investigation; writing—original draft; writing—review and editing; visualization. **William Robert Kwapong**: Conceptualization; methodology; software; data curation; investigation; validation; visualization; writing—original draft; writing—review and editing; formal analysis. **Biqiu Tang**: Investigation. **Junfeng Liu**: Investigation. **Wendan Tao**: Investigation. **Kun Lu**: Investigation; data curation. **Ruosu Pan**: Investigation; data curation. **Anmo Wang**: Investigation. **Lanhua Liao**: Investigation. **Tang Yang**: Investigation. **Le Cao**: Investigation; software; visualization. **Youjie Wang**: Investigation. **Shuai Jiang**: Investigation. **Xuening Zhang**: Investigation. **Ming Liu**: Funding acquisition; writing—review and editing; resources. **Bo Wu**: Funding acquisition; conceptualization; supervision; project administration; resources; writing—review and editing.

## CONFLICT OF INTEREST STATEMENT

The authors declare that they have no conflicts of interest.

### PEER REVIEW

The peer review history for this article is available at https://publons.com/publon/10.1002/brb3.3385.

## Supporting information

Supporting Information

## Data Availability

Data are available upon reasonable request from the corresponding author.
