# Supplementary material for: Association between functional network connectivity, retina structure and microvasculature, and visual performance in patients after thalamic stroke: An exploratory multi‐modality study
Source: Brain Behav. 2024 Jan 17;14(1):e3385. doi: 10.1002/brb3.3385 (PMC10794127; doi:10.1002/brb3.3385)
Supplement: Supplementary file 1 — Supporting Information [file BRB3-14-e3385-s001.docx]

Supplementary Materials

**Association between functional network connectivity, retina structure and microvasculature, and visual performance in patients after thalamic stroke: an exploratory multi-modality study**

*Supplementary figures*


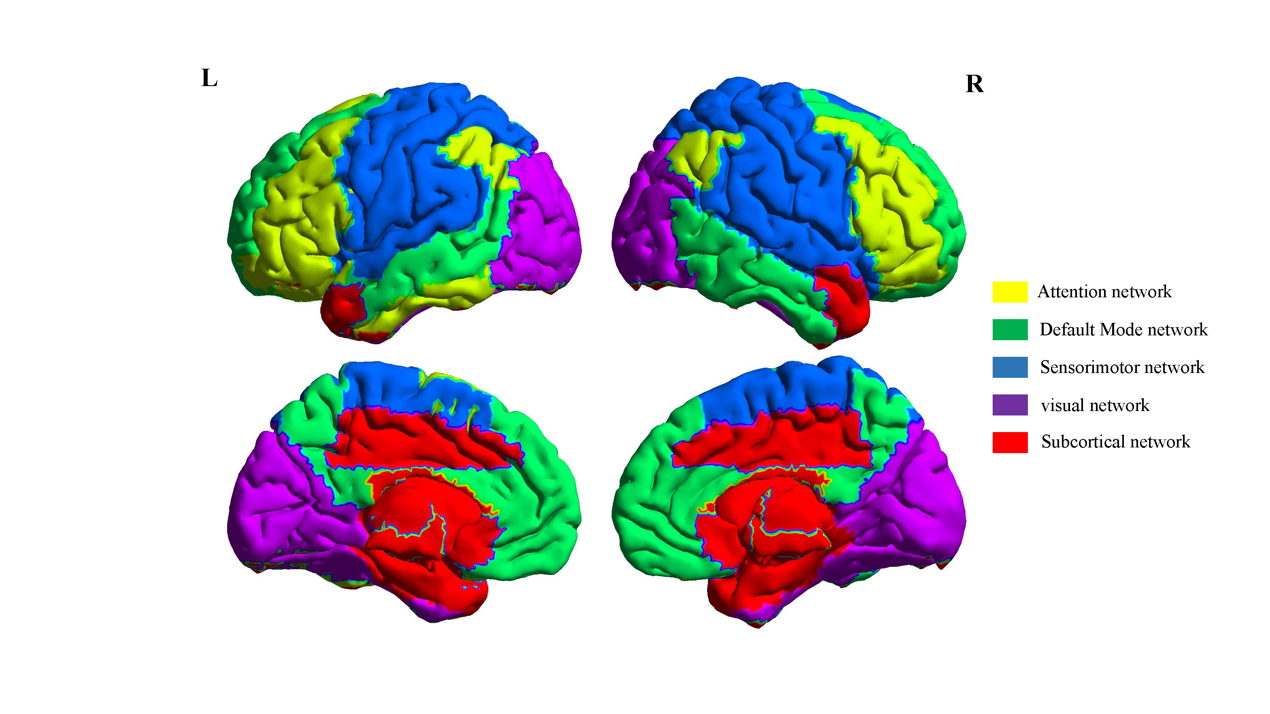


**Supplementary Figure 1.** Identified five empirical modules in the resting-state fMRI network. Defined as follows: Sensorimotor module (blue), Visual module (purple), Attention module (yellow), Default Mode module (green), and Subcortical module (red).

*Supplementary Tables*

**Supplementary Table 1.** The corresponding relationship of five resting-state functional network modules (Sensorimotor network, visual network, attention network, default mode network, and subcortical network) with nodes in Automated anatomical labelling atlas

| AAL_name | Acronyms | Module |  |
| --- | --- | --- | --- |
| Precentral_L | PreCG.L | Sensorimotor |  |
| Precentral_R | PreCG.R | Sensorimotor |  |
| Frontal_Sup_L | SFGdor.L | Default mode |  |
| Frontal_Sup_R | SFGdor.R | Default mode |  |
| Frontal_Sup_Orb_L | ORBsup.L | Attention |  |
| Frontal_Sup_Orb_R | ORBsup.R | Default mode |  |
| Frontal_Mid_L | MFG.L | Attention |  |
| Frontal_Mid_R | MFG.R | Attention |  |
| Frontal_Mid_Orb_L | ORBmid.L | Attention |  |
| Frontal_Mid_Orb_R | ORBmid.R | Attention |  |
| Frontal_Inf_Oper_L | IFGoperc.L | Attention |  |
| Frontal_Inf_Oper_R | IFGoperc.R | Attention |  |
| Frontal_Inf_Tri_L | IFGtriang.L | Attention |  |
| Frontal_Inf_Tri_R | IFGtriang.R | Attention |  |
| Frontal_Inf_Orb_L | ORBinf.L | Attention |  |
| Frontal_Inf_Orb_R | ORBinf.R | Attention |  |
| Rolandic_Oper_L | ROL.L | Sensorimotor |  |
| Rolandic_Oper_R | ROL.R | Sensorimotor |  |
| Supp_Motor_Area_L | SMA.L | Attention |  |
| Supp_Motor_Area_R | SMA.R | Sensorimotor |  |
| Olfactory_L | OLF.L | Subcortical |  |
| Olfactory_R | OLF.R | Subcortical |  |
| Frontal_Sup_Medial_L | SFGmed.L | Default mode |  |
| Frontal_Sup_Medial_R | SFGmed.R | Default mode |  |
| Frontal_Mid_Orb_L | ORBsupmed.L | Default mode |  |
|  |  |  |  |
| Frontal_Mid_Orb_R | ORBsupmed.R | Default mode |  |
|  |  |  |  |
| Rectus_L | REC.L | Default mode |  |
|  |  |  |  |
| Rectus_R | REC.R | Default mode |  |
|  |  |  |  |
| Insula_L | INS.L | Sensorimotor |  |
| Insula_R | INS.R | Sensorimotor |  |
| Cingulum_Ant_L | ACG.L | Default mode |  |
|  |  |  |  |
| Cingulum_Ant_R | ACG.R | Default mode |  |
|  |  |  |  |
| Cingulum_Mid_L | DCG.L | Subcortical |  |
| Cingulum_Mid_R | DCG.R | Subcortical |  |
| Cingulum_Post_L | PCG.L | Default mode |  |
| Cingulum_Post_R | PCG.R | Default mode |  |
|  |  |  |  |
| Hippocampus_L | HIP.L | Subcortical |  |
| Hippocampus_R | HIP.R | Subcortical |  |
| ParaHippocampal_L | PHG.L | Subcortical |  |
| ParaHippocampal_R | PHG.R | Subcortical |  |
| Amygdala_L | AMYG.L | Subcortical |  |
| Amygdala_R | AMYG.R | Subcortical |  |
| Calcarine_L | CAL.L | Visual |  |
| Calcarine_R | CAL.R | Visual |  |
| Cuneus_L | CUN.L | Visual |  |
| Cuneus_R | CUN.R | Visual |  |
| Lingual_L | LING.L | Visual |  |
| Lingual_R | LING.R | Visual |  |
| Occipital_Sup_L | SOG.L | Visual |  |
| Occipital_Sup_R | SOG.R | Visual |  |
| Occipital_Mid_L | MOG.L | Visual |  |
| Occipital_Mid_R | MOG.R | Visual |  |
| Occipital_Inf_L | IOG.L | Visual |  |
| Occipital_Inf_R | IOG.R | Visual |  |
| Fusiform_L | FFG.L | Visual |  |
| Fusiform_R | FFG.R | Visual |  |
| Postcentral_L | PoCG.L | Sensorimotor |  |
| Postcentral_R | PoCG.R | Sensorimotor |  |
| Parietal_Sup_L | SPG.L | Sensorimotor |  |
| Parietal_Sup_R | SPG.R | Sensorimotor |  |
| Parietal_Inf_L | IPL.L | Attention |  |
| Parietal_Inf_R | IPL.R | Attention |  |
| SupraMarginal_L | SMG.L | Sensorimotor |  |
| SupraMarginal_R | SMG.R | Sensorimotor |  |
| Angular_L | ANG.L | Attention |  |
| Angular_R | ANG.R | Attention |  |
| Precuneus_L | PCUN.L | Default mode |  |
| Precuneus_R | PCUN.R | Default mode |  |
| Paracentral_Lobule_L | PCL.L | Sensorimotor |  |
| Paracentral_Lobule_R | PCL.R | Sensorimotor |  |
| Caudate_L | CAU.L | Subcortical |  |
| Caudate_R | CAU.R | Subcortical |  |
| Putamen_L | PUT.L | Subcortical |  |
| Putamen_R | PUT.R | Subcortical |  |
| Pallidum_L | PAL.L | Subcortical |  |
| Pallidum_R | PAL.R | Subcortical |  |
| Thalamus_L | THA.L | Subcortical |  |
| Thalamus_R | THA.R | Subcortical |  |
| Heschl_L | HES.L | Sensorimotor |  |
| Heschl_R | HES.R | Sensorimotor |  |
| Temporal_Sup_L | STG.L | Sensorimotor |  |
| Temporal_Sup_R | STG.R | Sensorimotor |  |
| Temporal_Pole_Sup_L | TPOsup.L | Attention |  |
| Temporal_Pole_Sup_R | TPOsup.R | Sensorimotor |  |
| Temporal_Mid_L | MTG.L | Default mode |  |
| Temporal_Mid_R | MTG.R | Default mode |  |
| Temporal_Pole_Mid_L | TPOmid.L | Subcortical |  |
| Temporal_Pole_Mid_R | TPOmid.R | Subcortical |  |
| Temporal_Inf_L | ITG.L | Attention |  |
| Temporal_Inf_R | ITG.R | Default mode |  |

**Supplementary Table 2.** Association of multi-modular functional connectivity, and retina structural and microvascular characteristics, and visual acuity (p-values)

*Supplementary Table 2.1*

|  | Attention to Attention | Attention  To Default mode | Attention  To  Sensorimotor | Attention to Subcortical | Attention to Visual |
| --- | --- | --- | --- | --- | --- |
| SVC | 0.063 | **0.021** | 0.660 | 0.091 | 0.579 |
| DVC | 0.775 | 0.906 | 0.943 | 0.618 | 0.951 |
| RNFL | 0.783 | **0.010** | 0.562 | 0.378 | **0.026** |
| GCIPL | 0.287 | **0.036** | 0.850 | 0.393 | 0.106 |
| VA | 0.426 | 0.548 | 0.210 | 0.091 | 0.618 |

*Supplementary Table 2.2*

|  | Default mode to Default mode | Default mode to Sensorimotor | Default mode to Subcortical | Default mode to Visual | Sensorimotor to Sensorimotor | Sensorimotor to Subcortical |
| --- | --- | --- | --- | --- | --- | --- |
| SVC | 0.213 | 0.891 | 0.252 | 0.733 | 0.583 | 0.934 |
| DVC | 0.207 | 0.508 | 0.082 | 0.288 | 0.965 | 0.721 |
| RNFL | 0.096 | 0.641 | 0.344 | 0.267 | 0.433 | 0.454 |
| GCIPL | 0.490 | 0.908 | 0.145 | 0.903 | 0.585 | 0.528 |
| VA | 0.071 | 0.705 | 0.702 | 0.441 | 0.475 | 0.665 |

*Supplementary Table 2.3*

|  | Sensorimotor to Visual | Subcortical to Subcortical | Subcortical to Visual | Visual to Visual |
| --- | --- | --- | --- | --- |
| SVC | 0.054 | 0.086 | 0.545 | 0.089 |
| DVC | 0.257 | 0.307 | 0.969 | 0.131 |
| RNFL | 0.185 | 0.184 | 0.292 | **0.008** |
| GCIPL | 0.149 | 0.093 | 0.541 | **0.005** |
| VA | 0.596 | 0.337 | 0.342 | 0.675 |

SVC= superficial vascular complex; DVC= deep vascular complex; RNFL= retinal nerve fiber layer; GCIPL= ganglion cell and inner plexiform layer; VA= visual acuity, LogMAR. Adjusted for age, gender, hypertension, diabetes, dyslipidemia, disease duration, lesion volume.

Supplementary Table 3. Association of multi-modular functional connectivity, and retina structural and microvascular characteristics, and visual acuity in subgroup of disease duration≤6 months (p-values)

*Supplementary Table 3.1*

|  | Attention to Attention | Attention  To Default mode | Attention  To  Sensorimotor | Attention to Subcortical | Attention to Visual |
| --- | --- | --- | --- | --- | --- |
| SVC | 0.985 | 0.673 | 0.527 | 0.798 | 0.131 |
| DVC | 0.227 | 0.476 | 0.155 | 0.180 | **0.040** |
| RNFL | 0.879 | 0.438 | 0.554 | 0.324 | 0.803 |
| GCIPL | 0.835 | 0.889 | 0.637 | 0.462 | 0.508 |
| VA | 0.617 | 0.851 | 0.485 | **<0.001** | 0.145 |

*Supplementary Table 3.2*

|  | Default mode to Default mode | Default mode to Sensorimotor | Default mode to Subcortical | Default mode to Visual | Sensorimotor to Sensorimotor | Sensorimotor to Subcortical |
| --- | --- | --- | --- | --- | --- | --- |
| SVC | 0.688 | 0.743 | 0.921 | 0.249 | 0.442 | 0.267 |
| DVC | 0.154 | 0.682 | 0.098 | 0.366 | **0.006** | **0.014** |
| RNFL | 0.731 | 0.388 | 0.789 | 0.954 | 0.895 | 0.921 |
| GCIPL | 0.206 | 0.802 | 0.916 | 0.252 | 0.619 | 0.614 |
| VA | 0.128 | 0.869 | 0.168 | 0.792 | 0.216 | 0.431 |

*Supplementary Table 3.3*

|  | Sensorimotor to Visual | Subcortical to Subcortical | Subcortical to Visual | Visual to Visual |
| --- | --- | --- | --- | --- |
| SVC | 0.133 | 0.353 | 0.563 | 0.125 |
| DVC | 0.504 | **0.047** | 0.623 | 0.791 |
| RNFL | 0.760 | 0.977 | 0.634 | **0.020** |
| GCIPL | 0.701 | 0.674 | 0.934 | 0.013 |
| VA | 0.324 | 0.013 | 0.823 | 0.615 |

SVC= superficial vascular complex; DVC= deep vascular complex; RNFL= retinal nerve fiber layer; GCIPL= ganglion cell and inner plexiform layer; VA= visual acuity, LogMAR. Adjusted for age, gender, hypertension, diabetes, dyslipidemia, lesion volume.

Supplementary Table 4. Association between retina structural and microvascular characteristics and VA

|  | ß | SE | P |
| --- | --- | --- | --- |
| SVC | -10.979 | 4.249 | **0.013** |
| DVC | -2.666 | 3.275 | 0.420 |
| RNFL | -3.927 | 1.422 | **0.008** |
| GCIPL | -20.313 | 8.45 | **0.007** |

SVC= superficial vascular complex; DVC= deep vascular complex; RNFL= retinal nerve fiber layer; GCIPL= ganglion cell and inner plexiform layer; VA= visual acuity, LogMAR. Adjusted for age, gender, hypertension, diabetes, dyslipidemia, lesion volume.

Supplementary Table 5. Association of multi-modular functional connectivity, and retina structural and microvascular characteristics, and visual acuity in subgroup of disease duration>6 months (p-values)

*Supplementary Table 5.1*

|  | Attention to Attention | Attention  To Default mode | Attention  To  Sensorimotor | Attention to Subcortical | Attention to Visual |
| --- | --- | --- | --- | --- | --- |
| SVC | 0.193 | **0.008** | 0.213 | 0.375 | 0.260 |
| DVC | 0.465 | 0.227 | 0.480 | 0.278 | **0.003** |
| RNFL | 0.745 | 0.120 | 0.308 | 0.572 | 0.127 |
| GCIPL | 0.446 | **0.040** | 0.069 | 0.393 | 0.095 |
| VA | 0.236 | 0.541 | **0.017** | 0.531 | 0.411 |

*Supplementary Table 5.2*

|  | Default mode to Default mode | Default mode to Sensorimotor | Default mode to Subcortical | Default mode to Visual | Sensorimotor to Sensorimotor | Sensorimotor to Subcortical |
| --- | --- | --- | --- | --- | --- | --- |
| SVC | 0.420 | 0.593 | 0.623 | 0.573 | **0.042** | **0.035** |
| DVC | 0.731 | 0.342 | 0.102 | 0.592 | **0.025** | **0.002** |
| RNFL | 0.479 | 0.938 | 0.613 | 0.108 | 0.094 | 0.051 |
| GCIPL | 0.693 | 0.718 | 0.218 | 0.108 | **0.036** | **0.008** |
| VA | **0.039** | 0.309 | 0.552 | 0.742 | 0.771 | 0.790 |

*Supplementary Table 5.3*

|  | Sensorimotor to Visual | Subcortical to Subcortical | Subcortical to Visual | Visual to Visual |
| --- | --- | --- | --- | --- |
| SVC | 0.932 | 0.559 | 0.995 | 0.352 |
| DVC | 0.237 | 0.924 | 0.914 | 0.098 |
| RNFL | 0.298 | 0.426 | 0.152 | 0.180 |
| GCIPL | 0.319 | 0.352 | 0.660 | 0.245 |
| VA | 0.622 | 0.430 | 0.100 | 0.328 |

SVC= superficial vascular complex; DVC= deep vascular complex; RNFL= retinal nerve fiber layer; GCIPL= ganglion cell and inner plexiform layer; VA= visual acuity, LogMAR. Adjusted for age, gender, hypertension, diabetes, dyslipidemia, lesion volume.

Supplementary Table 6. Association between retina structural and microvascular characteristics and VA

|  | ß | SE | P |
| --- | --- | --- | --- |
| SVC | -1.772 | 5.857 | 0.765 |
| DVC | 2.000 | 3.599 | 0.523 |
| RNFL | 2.888 | 1.940 | 0.148 |
| GCIPL | -5.895 | 8.283 | 0.483 |

SVC= superficial vascular complex; DVC= deep vascular complex; RNFL= retinal nerve fiber layer; GCIPL= ganglion cell and inner plexiform layer; VA= visual acuity, LogMAR. Adjusted for age, gender, hypertension, diabetes, dyslipidemia, lesion volume.
